# Performance and safety of temperature- and flow-controlled radiofrequency ablation for ventricular arrhythmia

**DOI:** 10.1093/europace/euad372

**Published:** 2024-01-09

**Authors:** Richard Kobza, Gabriela Hilfiker, Sophie Rissotto, Saagar Mahida, Christian Grebmer, Firat Duru, Helmut Pürerfellner, Benjamin Berte

**Affiliations:** Heart Center, Luzerner Kantonsspital, Zentralstrasse 1, Lucerne 6004, Switzerland; Heart Center, Luzerner Kantonsspital, Zentralstrasse 1, Lucerne 6004, Switzerland; Clinical Support, Biosense Webster, Zug, Switzerland; Department of Cardiac Electrophysiology, Liverpool Heart and Chest Hospital, Liverpool, UK; Heart Center, Luzerner Kantonsspital, Zentralstrasse 1, Lucerne 6004, Switzerland; Cardiology Department, University Hospital Zürich, Zürich, Switzerland; Department of Cardiology, Ordensklinikum Linz Elisabethinen, Linz, Austria; Heart Center, Luzerner Kantonsspital, Zentralstrasse 1, Lucerne 6004, Switzerland

**Keywords:** Ventricular arrhythmia, Steam pop, Micro-EGM, Temperature guided

## Abstract

**Aims:**

High-power ablation is effective for ventricular arrhythmia ablation; however, it increases the risk of steam pops. The aim of this study was to define the safety and efficacy of QMODE ablation in the ventricle and the risk of steam pop.

**Methods and results:**

Consecutive patients undergoing ventricular ablation using QDOT were included in a prospective single-centre registry. Procedural data, complications, and follow-up were systematically analysed and compared with a historical ventricular tachycardia (VT) and premature ventricular complexes (PVC) cohort ablated using STSF. QMODE (≤50 W) ablation was performed in 107 patients [age 62 ± 13 years; 76% male; VT (*n* = 41); PVC (*n* = 66)]. A total of 2456 applications were analysed [power: 45.9 ± 5.0 W with minimal power titration (90% > 95% max power); duration 26 ± 8 s; impedance drop 9.4 ± 4.7 Ω; ablation index: 569 ± 163; mean–max temperature 44.3 ± 2.6°C]. Ventricular tachycardia ablation was associated with shorter radiofrequency (RF) time and a trend towards shorter procedure times using QDOT (QDOT vs. STSF: 20.1 ± 14.7 vs. 31 ± 17 min; *P* = 0.002, 151 ± 59 vs. 172 ± 48 min; *P* = 0.06). Complications, VT recurrence, and mortality rates were comparable (QDOT vs. STSF: 2% vs. 2%; *P* = 0.9, 24% vs. 27%; *P* = 0.82, and 2% vs. 4%; *P* = 0.67). Five audible steam pops (0.02%) occurred. Premature ventricular complex ablation was associated with comparable RF and procedure times (QDOT vs. STSF: 4.8 ± 4.6 vs. 3.9 ± 3.1 min; *P* = 0.25 and 96.1 ± 31.9 vs. 94.6 ± 24.7 min; *P* = 0.75). Complication and PVC recurrence were also comparable (QDOT vs. STSF: 0% vs. 3%; *P* = 0.17 and 19% vs. 22%; *P* = 0.71).

**Conclusion:**

Ventricular ablation using QMODE ≤ 50 W is safe and effective for both VT and PVC ablation and is associated with a low risk for steam pop.

## Graphical Abstract

**Graphical Abstract:**
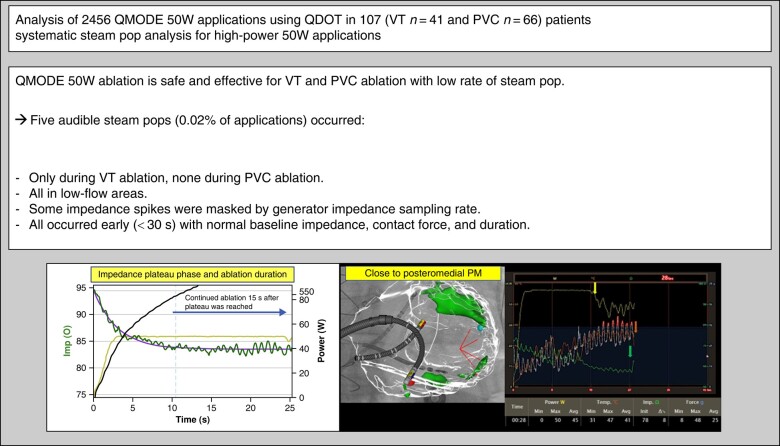

**What’s new?**

- High-power ablation using temperature- and flow-controlled radiofrequency ablation is safe and effective and results in a low risk of steam pop.
- Steam pop occurred in normal baseline impedance, normal contact force, and normal duration settings within low-flow areas in the ventricles.

## Introduction

While catheter ablation is a well-established treatment modality for patients with ventricular tachycardia (VT), complication rates remain high, and outcomes are still sub-optimal. Patient selection, procedure timing, standardization of endpoints, improved substrate definition, and more effective lesion creation, especially for intramural substrates, need to be optimized.^1^ A number of techniques have been developed for more effective lesion creation, including ablation with higher power and longer duration; use of low ionic solutions, such as half normal saline; and bipolar ablation.^2–5^ These approaches are designed to increase current density in tissue, which in turn leads to increased tissue temperature. However, the techniques also increase the risk of steam pop. It is important to note that while lesion formation using radiofrequency (RF) energy has been studied extensively in healthy tissue, biophysics of lesion formation in fatty and scarred tissue is less well defined. Previous animal studies have reported that, in scarred myocardium, lesion formation is unpredictable and lesions are more irregular.^6^

Recently, a novel irrigated catheter with incorporated thermocouples (QDOT, Biosense Webster Inc.) has been designed to provide more accurate measurement of the temperature at the catheter–tissue interface.^7^ Radiofrequency ablation using power-controlled mode with temperature feedback, including varying irrigation rates using this catheter, has the potential advantage of reducing the risk of steam pops.^8^ The potential disadvantage of the technology, however, is that power titration meant to prevent temperature from exceeding the desired maximum value could lead to smaller lesions, which in turn could adversely impact clinical effectiveness. In an *in vivo* animal study, lesion size was comparable with conventional ablation using this technology while the steam pop risk was reported to be lower.^8–10^ Of note, the true prevalence of steam pops during higher power longer duration ablation (e.g. 60–120 s) remains to be defined. The aim of this study was to evaluate the clinical performance of the QDOT catheter for ventricular arrhythmia ablation. We also sought to analyse the risk of steam pop using high-power longer duration ablation.

## Methods

Consecutive patients undergoing ablation of ventricular arrhythmias [VT/premature ventricular complexes (PVC)] using the QDOT catheter were included in a prospective single-centre registry. All patients provided informed consent. The study was approved by the local ethical committee (Ethikkommission Nordwest- und Zentralschweiz). Baseline characteristics, procedural data, procedure-related complications, and follow-up data were systematically collected. Catheter performance was investigated by the following parameters: arrhythmia recurrence during follow-up, risk of steam pop, amount of power titration, thermocouple temperature indication, RF time, and skin-to-skin time.

### Catheter design of QDOT compared with STSF

The QDOT catheter is based on the SmartTouch SF catheter platform. It has an irrigated 3.5 mm tip electrode with three additional micro-electrodes (0.086 mm^2^ surface; 1.5 mm inter-electrode distance; 120° angle orientation) and six embedded thermocouples (sets of three; 1 mm diameter; 120° angle orientation). The electrodes are designed to improve near-field electrogram (EGM) signal quality. The thermocouples are designed to provide real-time feedback of tissue surface heating, as a proxy for the lesion core temperature. Based on the design, the catheter can be used in power-controlled mode (QMODE up to 50 W; QMODE+ for 90 W ablation) with temperature feedback, presented in a 2D window on the CARTO system (also referred to as the ‘bullseye’). In comparison, the SmartTouch SF catheter is an open-irrigated 3.5 mm tip catheter with 56 porous holes and 1 embedded thermocouple. This thermocouple measures the cooled tip temperature and not the tissue surface temperature. This catheter is typically used in a power-controlled mode.^11^

### Ablation procedure using QMODE

QDOT catheter ablation was performed in the QMODE setting (≤50 W) with baseline impedance around 100 Ω (single indifferent electrode). The typical ablation duration was 25–40 s, with prolongation to 120 s for intramural substrates. The target impedance drop was ≥10 Ω. Ablation performed in QMODE allows the ablation to be adjusted through a combination of flow adaptation and power titration, in order to keep the temperature in a pre-defined range. By setting a target temperature to 45°C with a maximum low-flow temperature value of 40°C, the ablation is performed at 50 W, as long as the irrigation flow rate is able to keep the measured temperature within this range. When the higher irrigation alone is insufficient to affect the temperature response, the power delivered is decreased (power titration), therefore reducing the temperature.^12^

### Ventricular tachycardia ablation using QDOT

Ventricular tachycardia ablation was performed using an image-guided, substrate-based approach in sinus rhythm as outlined in previous studies.^13^ Access to the left ventricle was gained using a combined retrograde aortic and antegrade transseptal route. A large-curve steerable sheath (Vizigo 8.5F, Biosense Webster, Inc.), bidirectional QDOT catheter (F-J curve), and multipolar mapping catheter (PentaRay F-curve or OctaRay F-curve, Biosense Webster, Inc.—at operator’s discretion) were used for VT ablation cases. All audible steam pops (together with the anatomical location of the steam pop) were prospectively documented.

### Comparison with ventricular tachycardia ablation using STSF

We compared safety and efficacy with conventional ablation (SmartTouch SF catheter, Biosense Webster, Inc.) using a retrospective cohort from the two-centre MUSIC VT study as the control arm (performed between January 2017 and March 2018). Of note, the MUSIC VT study involved the same operators and an identical dechanneling protocol for VT ablation, except for the catheter used during ablation (control cohort: MUSIC-guided VT ablation; detailed mapping using dedicated mapping catheter; conventional 45 W and 60 s applications).^13^

### Premature ventricular complex ablation using QDOT

**Figure 1:**
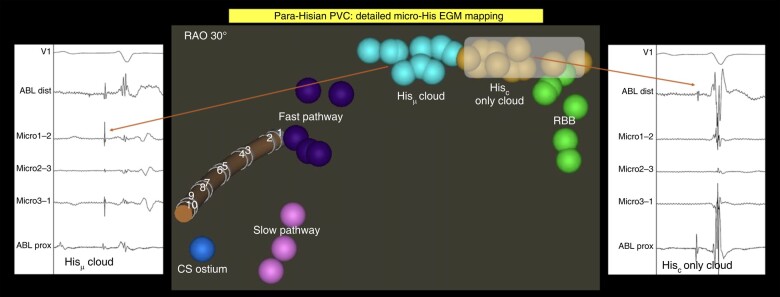
Para-Hisian PVC strategy using micro-His cloud avoidance strategy. Adapted from Berte *et al.*, Heart Rhythm 2022 ‘High-resolution para-Hisian mapping and ablation using micro-electrode embedded ablation catheters’ with permission from the author. *Left:* Micro-His EGM with clear His EGM on both conventional electrodes and micro-electrodes (arrows). *Middle:* Light blue, micro-His cloud that should be avoided during ablation to avoid AV block; yellow, macro-only His cloud where prudent ablation is possible. *Right:* Absence of a His signal on the micro-electrodes. Presence of His signals on the conventional electrodes.

Left ventricular access was gained using a combined retrograde aortic and antegrade transseptal route. A non-steerable sheath [Preface (8F), Biosense Webster, Inc.] was used for right ventricular and coronary sinus mapping/ablation. A bidirectional QDOT catheter (D–F-curve) was used for all cases. A PentaRay (F-curve) or OctaRay (F-curve) catheter (Biosense Webster, Inc.) was used for mapping at the operators’ discretion. The site of origin was identified based on activation mapping (QS unipolar EGM; pre-QRS onset with earliest micro-electrode EGM signals) and pace mapping (≥98% match). All audible steam pops (together with the anatomical location of the steam pop) were prospectively documented. For para-Hisian PVC ablation, the ablation strategy involved creation of a high-density macro- and micro-His cloud followed by avoidance of the micro-His cloud as described earlier^14^ (*Figure 1*).

### Comparison with premature ventricular complex ablation using STSF

We compared safety and efficacy of ablation with conventional ablation (SmartTouch SF catheter, Biosense Webster Inc.) using a retrospective cohort from our patients that were included in the Swiss PVC registry as the control arm (performed from October 2015 till November 2017).^15^

### Analysis of steam pops

Data on all audible steam pops (including the location of steam pops) were systematically and prospectively documented. In order to ensure that no data was missing, a Python script was written to retrospectively cross-check all cases and retrieve those in which an event of audible steam pop was recorded during ventricular arrhythmia ablation performed with QDOT. All applications were checked in the CARTO ablation export file of all cases and screened for the keywords ‘pop’, ‘steam pop’, and ‘steam-pop’ to cross-check data against the prospectively collected data in the electronic medical record (EPIC). All procedures with steam pop were reviewed, and the location and ablation data of the pop were analysed in detail. If the inHEART model was available, the location of the valves and papillary muscles in relation to the site of steam pop was documented. Applications leading to a steam pop were compared with applications without audible steam pop (within the same patient) to detect differences in temperature dynamics, power setting, contact force (CF), catheter orientation, location, baseline impedance, impedance drop, impedance plateau phase, and ablation duration.

### Analysis of lesion parameters

**Figure 2:**
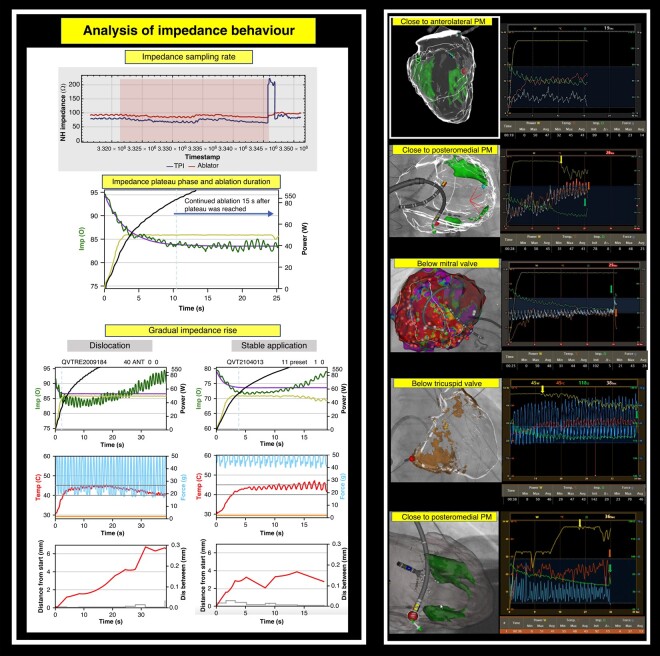
Steam pop analysis. *From left to right and top to bottom. Upper left:* Example of a steam pop using the Navistar 4 mm catheter during AVNRT. The impedance spike is not visible on the graph due to a lower impedance sampling rate of the generator. Retrospective analysis of the raw data impedance did demonstrate a clear typical fast and high impedance rise during the steam pop. No gradual impedance rise was seen before the pop. *Low left:* analysis of all RF application for impedance drop, impedance plateau phase, and application duration. *Upper right:* Five steam pops (0.3% of VT applications and 11% of VT procedures) during QMODE 50 W ablation with the QDOT catheter. (1) Superior glass mode view of inHEART model during VT ablation of anterolateral scar. Papillary muscles are in green. Red tag is application that resulted in steam pop. Vizigo sheath, DECANAV, and QDOT catheter visible. (2) Right anterior oblique view of a patient after inferior infarction with post-ischaemic posterior papillary muscle (in green on inHEART model). Red tag is application that resulted in steam pop. Vizigo sheath, DECANAV, and QDOT catheter visible. (3) Ventricular tachychardia ablation due to electrical storm. Patient after large inferior myocardial infarction. Bipolar voltage map (0.5–1.5 mV) of a large inferior substrate after myocardial infarction. Steam pop point is highlighted with orange halo around visitag, curved catheter below the mitral valve. DECANAV, PentaRay, and QDOT catheters (all Biosense Webster) are visible. (4) Arrhythmogenic right ventricular cardiomyopathy ablation below the lower part of the tricuspid valve. inHEART model in Glassmode in AP view with fibrofatty replacement substrate in brown. Red dot = ablation tag that resulted in steam pop. (5) Right anterior oblique view of a patient after inferior infarction with post-ischaemic posterior papillary muscle (in green on inHEART model). Red tag is application that resulted in steam pop. Vizigo sheath, PentaRay catheter, and QDOT catheter visible. *Right side:* Graphs during corresponding applications. Power in yellow, impedance in green, maximal thermocouple temperature in orange, and contact force in blue. In none of the graphs, there was a gradual impedance rise before the pop. (1) Absence of prior power titration. Absence of impedance spike during steam pop, due to lower sampling rate as described above. (2) Power titration (yellow arrow) due to insufficient cooling after increased irrigation. Impedance spike (green arrow) during steam pop and temperature drop. (3) Absence of prior power titration. Impedance spike (green arrow) during steam pop and temperature drop (orange arrow). (4) Power titration (yellow arrow) due to insufficient cooling after increased irrigation. Impedance spike (green arrow) during steam pop and temperature drop. (5) Power titration (yellow arrow) due to insufficient cooling after increased irrigation. Impedance spike (green arrow) during steam pop and temperature drop.

All RF applications were retrospectively analysed for impedance changes, CF, catheter orientation, temperature, and power titration. The impedance curve was fit to an exponential decay model, and its exponential time constant (Tau) was used to evaluate the speed of decay of each application. For all curves, the final plateau value was defined as 97% of the overall decay. The time to reach the plateau was then computed, and the duration of ablation after the plateau phase was reached was analysed in seconds (*Figure 2*). If the plateau phase was detected within 2.5 s or later than 120 s, the application was excluded from further analysis. For all other applications, it was noted if the plateau phase was reached within 25 s and 40 s or not reached.

### Statistical analysis

Data analysis was performed using SPSS Statistics (IBM, version 24). Normality of data was assessed using the Shapiro–Wilk test. Continuous variables were expressed as mean **±** standard deviation or median (interquartile range). Comparison of means between groups was performed using the independent sample *t*-test for normally distributed data and the Mann–Whitney *U* test for non-uniformly distributed data. The χ^2^ test was used to compare categorical data. A *P* value < 0.05 was considered to be statistically significant.

## Results

### Baseline characteristics

Between December 2019 and December 2021, 107 consecutive patients undergoing ablation of ventricular arrhythmias using the QDOT catheter (QMODE; ≤50 W; temperature cut-off 45°C) at the Luzerner Kantonsspital were included. Sixty-six (61%) underwent a PVC ablation and 41 (39%) underwent VT ablation (ischaemic cardiomyopathy, 51%; dilated cardiomyopathy, 10%; arrhythmogenic right ventricular cardiomyopathy, 10%). In comparison with patients undergoing PVC ablation, those undergoing VT ablation were of comparable age (PVC vs. VT: age, 60.8 ± 13.2 vs. 64.3 ± 10.8, *P* = 0.16) and had a lower left ventricular ejection fraction (PVC vs. VT: age, LVEF, 41% vs. 54%, *P* < 0.001). As summarized in *Table 1*, the VT ablation group also had a higher prevalence of diabetes mellitus, hypertension, renal insufficiency, and atrial fibrillation.

**Table 1:** Baseline characteristics

	VT	PVC	*P* value
Total number	41	66	< 0.05
Age (years)	64.3 ± 10.8	60.8 ± 13.2	0.16
Male sex (*n*, %)	36 (88%)	47 (71%)	0.06
BMI (mean ± SD)	28.3 ± 5.3	27.1 ± 4.2	0.2
Hypertension (*n*, %)	25 (61%)	31 (47%)	0.18
DM type II (*n*, %)	10 (24%)	6 (9%)	0.03
History of smoking (*n*, %)	12 (29%)	17 (26%)	0.73
Dyslipidaemia (*n*, %)	17 (41%)	24 (36%)	0.64
Family history of CHD (*n*, %)	7 (17%)	14 (21%)	0.57
History of stroke (*n*, %)	6 (15%)	1 (2%)	<0.005
Structural heart disease (*n*, %)	33 (80%)	16 (24%)	<0.001
Ischaemic cardiomyopathy (*n*, %)	21 (51%)	13 (19.7%)	<0.001
Hypertrophic cardiomyopathy (*n*, %)	1 (2%)	0	
Dilated cardiomyopathy (*n*, %)	4 (10%)	0	
ARVC (*n*, %)	4 (10%)	0	
Other (*n*, %)	3 (7%)	3 (4.5%)	0.56
PVC-induced CMP (*n*, %)	8 (20%)	2 (3%)	<0.005
LVEF (%)	41.4 ± 10.6	53.6 ± 8.8	<0.001
GFR < 60 (*n*, %)	11 (27%)	7 (11%)	0.03
Severe MI (*n*, %)	2 (4.9%)	2 (3%)	0.64
History of AF (*n*, %)	15 (36.6%)	10 (15%)	0.01
PVC burden (%)	NA	23 ± 10%	
Device therapy (*n*, %)	22 (53%)	3 (4.5%)	<0.001
Pacemaker (*n*, %)	1 (2%)	0	
ICD (*n*, %)	18 (44%)	2 (3%)	<0.001
CRT (*n*, %)	3 (7%)	1 (2%)	0.13
First procedure (*n*, %)	39 (59%)	64 (97%)	0.31
Redo procedure (*n*, %)	4 (41%)	2 (3%)	0.15
AAD			
Beta-blocker (*n*, %)	27 (66%)	40 (61%)	0.65
Sotalol or flecainide (*n*, %)	3 (7%)	3 (4.5%)	0.56
Amiodarone (*n*, %)	7 (17%)	36 (55%)	<0.001
NOAC (*n*, %)	11 (27%)	9 (14%)	0.1
Vit K antagonist (*n*, %)	6 (15%)	5 (8%)	0.25

AAD, anti-arrhythmic drugs; AF, atrial fibrillation; ARVC, arrhythmogenic right ventricular cardiomyopathy; BMI, body mass index; CHD, coronary heart disease; CMP, cardiomyopathy; CRT, cardiac resynchronization therapy; DM, diabetes mellitus; GFR, glomerular filtration rate; ICD, intracardiac cardioverter; LVEF, left ventricular cardiomyopathy; MI, mitral insufficiency; NOAC, novel oral anticoagulants; PVC, premature ventricular complex; SD, standard deviation; vit, vitamin; VT, ventricular tachycardia.

### Ventricular tachycardia ablation

#### Procedural data

**Figure 3:**
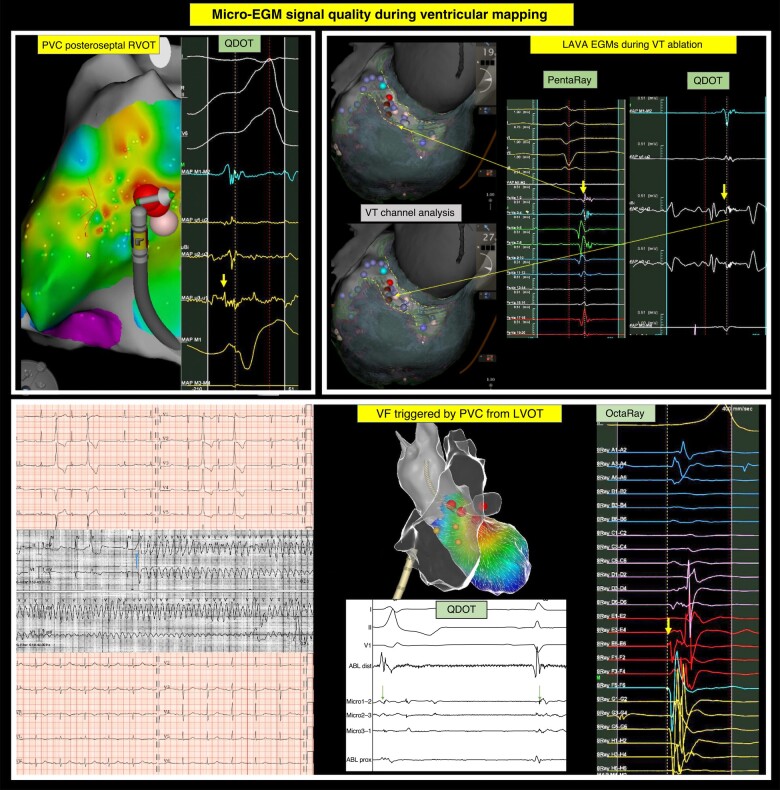
Micro-EGM signal quality during PVC, VT, and VF ablation. *From left to right and top to bottom. Upper left:* Posteroanterior view of activation map (CARTO) during the ablation of a posteroseptal RVOT PVC. QDOT demonstrates very nice fragmentation on micro-EGM (yellow arrow). Earliest activation was manually readapted towards first peak of micro-EGM activation. Premature ventricular complex was gone after 2.7 s QMODE 50 W ablation. *Upper right:* Posteroanterior view of inHEART model with inferior scar and clear CT channel. Within the CT channel, LAVA activity was found. At exactly the same location, similar EGM was found of the PentaRay catheter and the micro-electrodes of the QDOT catheter (yellow arrows). *Lower panel:* Trigger ablation for ventricular fibrillation. Upper ECG: dynamic LVOT PVC coupling as a sign of ischaemia. Middle telemetry: documented ventricular fibrillation due to early PVC within the vulnerable phase of the T-wave. Lower ECG: Absence of PVC, sinus rhythm. The earliest activity was first mapped using the OctaRay catheter (Biosense Webster) with good differentiation between sharp near-field activity (yellow arrow) and blunt far-field activity. Earliest activity was found in the left coronary cusp (LCC) and LVOT region. At the ablation site, subtle early fragmentation was seen during PVC and late fragmentation during sinus rhythm on the micro-electrodes.

Procedural data and a representative example of micro-EGM-guided VT ablation are included in *Table 2* and *Figure 3*, respectively. The overall procedure duration was 149 ± 60 min with an ablation time of 25.1 ± 12.3 min. The average CF was 18.4 ± 11.0 g, maximum power was 49.6 ± 3.4 W, and ablation duration averaged 25.1 ± 12.3 s. The mean impedance drop was 9.1 ± 4.0 Ω with an ablation index (AI) of 585 ± 149. Power titration was rare and short-lived with 90% of the time >95% max power, for an average max temp of 44.4 ± 2.4°C, resulting in a mean power of 46.3 ± 4.3 W (*Table 3*). The impedance plateau was reached in ≤25 s for 81% of applications; at ≤40 s, the proportion increased to 85%. A total of 181 applications (11%) were >40 s.

**Table 2:** Procedural data and follow-up

	VT	PVC	*P* value
Skin-to-skin time (min)	151 ± 59	96.1 ± 31.9	<0.001
Fluoroscopy time (min)	6.6 ± 4.8	4.3 ± 3.0	0.02
RF time (min)	20.1 ± 14.7	4.8 ± 4.6	<0.001
Ablation time (min)	65.5 ± 33.0	37.9 ± 30.2	<0.001
Anaesthesia standby or GA (*n*, %)	28 (68%)	4 (6%)	<0.001
Dedicated mapping catheter (*n*, %)	34 (82.9%)	15 (23%)	<0.001
Combined TS + R approach (*n*, %)	28 (68.3%)	2 (3%)	<0.001
Non-inducibility of any VT (*n*, %)	34 (82.9%)	NA	
Major complications (*n*, %)	1 (2%)	0	0.02
Audible steam pop (*n*, %)	5 (12%)	0	<0.001
Tamponade (*n*, %)	1 (2%)	0	0.20
Stroke	0	0	
Vascular bleeding	0	0	
Death (*n*, %)	1 (2%)	0	0.20
VT recurrence during FU (*n*, %)	10 (24%)	NA	
Death during FU (*n*, %)	1 (2%)	0	0.20
Duration of FU (mo)	12 ± 16	12 ± 9	1
PVC recurrence during FU (*n*, %)	NA	13 (19%)	
PVC burden during FU (%)	NA	7.7 ± 10%	

FU, follow-up; GA, general anaesthesia; NA, not applicable; PVC, premature ventricular complex; R, retrograde; RF, radiofrequency; SD, standard deviation; TS, transseptal; VT, ventricular tachycardia.

**Table 3:** Ablation data

	VT	PVC	*P* value
Ablation duration pro application (s)	25.13 ± 12.33	24.82 ± 16.81	0.68
Ablation duration >40 s (%)	11.9	19.3	<0.001
Average ablation index	585 ± 149	523 ± 194	<0.001
Average baseline imp (Ohms)	101.6 ± 15.9	118.8 ± 18.7	<0.001
Average imp drop during application	9.2 ± 4.0	10.4 ± 6.4	<0.001
Time to plateau phase (s)	16.0 ± 14.5	17.7 ± 21.1	0.09
Average contact force (g)	18.4 ± 11.0	14.8 ± 10.8	<0.001
Average maximal temp (°)	44.4 ± 2.4	44.0 ± 3.3	0.01
Average power after reaching 95% of max power (W)	48.5 ± 4.3	46.6 ± 6.3	<0.001
Average Tau (s)	4.5 ± 4.1	5.1 ± 6.0	0.09
Imp plateau phase reached within 25 s (%)	80.7	77.3	0.11
Imp plateau phase reached within 40 s (%)	85.4	83.3	0.26
Continued ablation duration after plateau (%)	85.4	83.3	0.26

app, application; imp, impedance; PVC, premature ventricular complex; s, seconds; VT, ventricular tachycardia; W, watts.

#### Procedural complications

In total, 5 steam pops were observed in 1678 (0.3%) applications in 41 patients who underwent VT ablation. All steam pops were observed in low-flow areas adjacent to the atrioventricular (AV) valve apparatus [below mitral valve (*n* = 1), below tricuspid valve (*n* = 1), adjacent to papillary muscles (*n* = 3); *Figure 2*]. Power titration occurred in 60% of steam pops. Gradual impedance rises were not observed prior to any of the steam pops. Temperature drops were consistently seen during the steam pops. During one steam pop, an impedance spike was not seen due to a lower impedance sampling rate of the generator with respect to the graph visualized (*Figure 2*). Applications resulting in a steam pop had a steeper impedance decrease and lower final impedance but a longer time to plateau phase. The baseline impedance and application duration were comparable with lesions without steam pops (no steam pop vs. steam pop: 97.9 ± 14.0 vs. 96.0 ± 1.2 Ω; *P* = 0.61 and 25.1 ± 12.0 vs. 29.0 ± 7.3 s; *P* = 0.47). Steam pops were associated with a higher CF and lower maximal temperature (no steam pop vs. steam pop: CF: 20.2 ± 1.8 vs. 24.9 ± 11.8 g; *P* < 0.001; temperature: 49.5° ± 0.7° vs. 46.0° ± 1.2°; *P* < 0.001). After the plateau phase was reached, ablation was continued for a shorter period in the steam pop group (no steam pop vs. steam pop: 13.5 ± 3.7 vs. 6.9 ± 8.2 s; *P* < 0.001; *Table 4*).

**Table 4:** Steam pop analysis (within-case analysis)

	No steam pop	Steam pop	*P* value
Total number of steam pop (*n*, %)	NA	5 (0.3% app/11% pr)	
Time to plateau phase (s)	16.3 ± 2.9	30.4 ± 18.6	<0.001
Average contact force (g)	20.2 ± 1.8	24.9 ± 11.8	<0.001
Average maximal temp (°)	49.5 ± 0.7	46.0 ± 1.2	<0.001
Baseline imp (Ohms)	97.9 ± 14.0	101.1 ± 9.2	0.61
Imp drop (Ohms)	9.7 ± 1.3	14.4 ± 3.8	<0.001
Average Tau (s)	4.65 ± 4.62	8.69 ± 5.30	0.05
Ablation after reaching plateau (s)	13.5 ± 3.7	6.9 ± 8.2	<0.001
Time of ablation/time to pop (s)	25.1 ± 12.0	29.0 ± 7.3	0.47
Gradual impedance rise	Not known	0 (0%)	

app, applications; imp, impedance; NA, not applicable; pr, VT procedures; s, seconds.

In one patient, the QDOT catheter was entrapped in the mitral valve apparatus (retrograde aortic left ventricular access). The catheter was eventually retracted using constant tension, 30 mL irrigation, and a QMODE plus 90 W ablation. Moderate mitral regurgitation was observed post-procedure, without evidence of a flail leaflet. There were no strokes or vascular complications. One patient had a pericardial tamponade requiring percutaneous drainage (in the absence of an audible steam pop).

#### Follow-up

After a mean follow-up of 12 ± 16 months, 10 (24%) patients had VT recurrences. Two patients died during follow-up: one patient died 1 week post- ablation due to a fulminant COVID-19 infection and one patient died due to valve thrombosis during extracorporeal membrane oxygenation (ECMO) treatment 3 weeks post-ablation.

#### Retrospective comparison with the MUSIC VT study

We compared the cohort undergoing VT ablation using the QDOT catheter to a retrospective cohort which underwent VT ablation with a conventional ablation catheter (SmartTouch catheter). Of note, both cohorts had an identical image-guided ablation protocol. As demonstrated in *Table 5*, baseline characteristics were comparable between the two cohorts. QDOT ablation was associated with shorter ablation time (QDOT vs. conventional: 20.1 ± 14.7 vs. 31 ± 17 min; *P* = 0.002) and a trend towards shorter procedure times (QDOT vs. conventional: 151 ± 59 vs. 172 ± 48 min; *P* = 0.06). Complication (QDOT vs. conventional: 2% vs. 2%; *P* = 0.9), VT recurrence rates (QDOT vs. conventional: 24% vs. 27%; *P* = 0.82), and mortality rates (QDOT vs. conventional: 2% vs. 4%; *P* = 0.67) were comparable.

**Table 5:** Retrospective comparison between STSF and QDOT for VT ablation

	QDOT	STSF	*P* value
Total number	41	49	
Age (years)	64.3 ± 10.8	63 ± 15	0.64
Male sex (*n*, %)	36 (88%)	44 (90%)	0.76
BMI (mean ± SD)	28.3 ± 5.3	26.6 ± 3.7	0.08
Hypertension (*n*, %)	25 (61%)	29 (59%)	0.86
DM type II (*n*, %)	10 (24%)	4 (8%)	0.03
History of smoking (*n*, %)	12 (29%)	20 (41%)	0.25
Ischaemic cardiomyopathy (*n*, %)	21 (51%)	34 (69%)	0.08
Dilated cardiomyopathy (*n*, %)	4 (10%)	10 (20%)	0.16
ARVC (*n*, %)	4 (10%)	2 (4%)	0.28
LVEF (%)	41.4 ± 10.6	41 ± 14	0.88
GFR < 60 (*n*, %)	11 (27%)	5 (10%)	0.04
ICD (*n*, %)	18 (44%)	37 (76%)	0.002
CRT (*n*, %)	3 (7%)	4 (8%)	0.88
Beta-blocker (*n*, %)	27 (66%)	45 (92%)	0.002
Sotalol or flecainide (*n*, %)	3 (7%)	3 (4.5%)	0.82
Amiodarone (*n*, %)	7 (17%)	19 (39%)	0.02
Skin-to-skin time (min)	151 ± 59	172 ± 48	0.06
RF time (min)	20.1 ± 14.7	31 ± 17	0.002
Ablation time (min)	65.5 ± 33.0	79.0 ± 37.0	0.07
Dedicated mapping catheter (*n*, %)	34 (82.9%)	37 (76%)	0.39
Combined TS + R approach (*n*, %)	28 (68.3%)	26 (53%)	0.14
Major complications (*n*, %)	1 (2%)	1 (2%)	0.9
Minor complications (*n*, %)	1 (2%)	0	0.89
Audible steam pop (*n*, %)	5 (12%)	Not known	
Death during FU (*n*, %)	2 (4.8%)	2 (4%)	0.86
FU duration (mo)	12 ± 16	19 ± 8	0.02
VT recurrence during FU (*n*, %)	10 (24%)	13 (27%)	0.82

ARVC, arrhythmogenic right ventricular cardiomyopathy; BMI, body mass index; CRT, cardiac resynchronization therapy; DM, diabetes mellitus; FU, follow-up; GFR, glomerular filtration rate; ICD, intracardiac cardioverter; LVEF, left ventricular ejection fraction; *n*, number; R, retrograde; RF, radiofrequency; SD, standard deviation; TS, transseptal; VT, ventricular tachycardia.

### Premature ventricular complex ablation

#### Procedural data

Procedural and follow-up data are presented in *Table 2*. Micro-EGM examples during PVC ablation are included in *Figure 3*. Procedure duration was 96 ± 32 min with RF time 3.9 ± 2.9 min. The average CF was 14.8 ± 10.8 g with a maximum power of 47.75 ± 5.66 W and an ablation duration of 34.8 ± 16.8 s. The impedance drop was 10.4 ± 6.4 Ω with an AI of 523 ± 194. Power titration was rare and short-lived with 90% of the time > 95% max power for an average max temp of 44.0 ± 3.3°C, resulting in a mean power of 44.4 ± 6.6 W. The impedance plateau was reached in ≤25 s for 77% of lesions; at ≤40 s, the proportion increased to 83%. Overall, 114 applications (19%) were > 40 s. The PVCs were ablated from the following anatomical locations: right ventricular outflow tract (RVOT) [*n* = 23 (35%)], left ventricular outflow tract (LVOT) [*n* = 17 (26%)], distal cardiac vein [*n* = 2 (3%)], left summit [*n* = 4 (6%)], para-Hisian [*n* = 8 (12%)], and papillary muscles [*n* = 8 (12%)] and anterolateral (*n* = 5), posteromedial (*n* = 3), aortic cusps [*n* = 5 (8%)], and moderator band [*n* = 2 (3%)].

#### Procedure complications and follow-up

No complications or steam pops occurred during PVC ablation. After a mean follow-up of 12 ± 16 months, 13 (19%) PVC recurrences were observed. There were no deaths following PVC ablation (*Table 6*).

**Table 6:** Retrospective comparison between STSF and QDOT for PVC ablation

	QDOT	STSF	*P* value
Total number	66	35	
Age (years)	60.8 ± 13.2	53.3 ± 14.0	<0.001
Male sex (*n*, %)	47 (71%)	17 (49%)	0.03
Ischaemic cardiomyopathy (*n*, %)	13 (19.7%)	1 (3%)	0.02
Dilated cardiomyopathy (*n*, %)	0	0	
PVC-induced CMP (*n*, %)	2 (3%)	3 (9%)	0.22
Outflow tract PVC	56 (85%)	35 (100%)	0.02
LVEF (%)	53.6 ± 8.8	60.0 ± 9	0.001
ICD (*n*, %)	2 (3%)	1 (3%)	0.96
CRT (*n*, %)	1 (2%)	0	0.46
Class II or IV (*n*, %)	40 (61%)	25 (71%)	0.28
Class Ic or III (*n*, %)	39 (59%)	12 (37%)	0.02
Skin-to-skin time (min)	96.1 ± 31.9	94.6 ± 24.7	0.79
RF time (min)	4.8 ± 4.6	3.9 ± 3.1	0.25
Ablation time (min)	37.9 ± 30.2	Not known	NA
Major complications (*n*, %)	0	1 (3%)	0.17
Audible steam pop (*n*, %)	0	0	
Death during FU (*n*, %)	0	0	
FU duration (months)	12 ± 9	6 ± 8	<0.001
PVC recurrence during FU (*n*, %)	13 (19%)	8 (22%)	0.71
PVC burden during FU (%)	7.7 ± 10%	4.7 ± 8.5%	0.14

CMP, cardiomyopathy; CRT, cardiac resynchronization therapy; FU, follow-up; ICD, intracardiac cardioverter; LVEF, left ventricular ejection fraction; *n*, number; PVC, premature ventricular complex; RF, radiofrequency.

## Discussion

The main findings of this study are the following: ablation of ventricular arrhythmia using QMODE 50 W is associated with (i) a very low incidence of steam pops, (ii) a low risk of complications, (iii) minimal power titration, (iv) shorter RF time and a trend towards shorter procedure times compared with conventional ablation, and (v) the safety and efficacy of QMODE 50 W ablation is comparable with standard ablation.

### Power titration: limited but effective

In the present study, power titration was observed in a third of lesion applications. Of note however, in most of these lesions, power titration occurred during <10% of the overall ablation duration. Furthermore, the power attenuation was subtle with an average power reduction of −5 W. These findings are in keeping with the observations during atrial QMODE 50 W ablation.^12^ Anter *et al.*^9^ previously reported that the power titration associated with QMODE ablation lowers the surface temperature and therefore reduces the risk of steam pop.

### Steam pop risk during higher power longer duration ablation (50 W)

Using a thigh preparation, Anter and colleagues previously reported a high incidence of steam pops (80%) following high-power ablation (50 W) using an STSF catheter. In comparison, temperature feedback (QMODE 50 W) was associated with a significantly lower incidence of steam pops [35% (*T*_max_ 50°C) and 0% (*T*_max_ 45°C)]. Furthermore, in keeping with our findings, in a beating ventricle, they identified a 7% (*T*_max_ 45°C) incidence of steam pops.^9^ For both atrial and ventricular ablation, we used a *T*_max_ cut-off of 45°C. We identified steam pops in 12% of VT cases. The lower measured thermocouple temperature during the steam pop occurrence is hypothesized to be the result of a mechanical dislocation due to the local explosion. As previously discussed, QMODE behaviour initially increases flow to a maximum of 15 mL/min and subsequently reduces power if necessary, as observed in three out of five steam pops. All steam pops occurred in low-flow areas after an ablation duration between 25 and 40 s. Therefore, despite limiting the ablation duration in these regions, we were unable to prevent these steam pops. It is however unclear if overall additional steam pops were prevented by limiting the ablation duration. Interestingly, we did not identify abnormal lesion parameters, including impedance rises, prior to steam pops. Of note, steam pops occurred exclusively in patients undergoing VT ablation (rather than PVC ablation). We hypothesize that apart from PVC ablation cases involving the papillary muscles, the risk of embedding the catheter tip within the tissue in a low-flow area is lower during PVC ablation.

### Incidence of steam pops

There is a relative paucity of data on the incidence of steam pops during ablation of ventricular tachycardia. Seiler *et al.*^16^ analysed the prevalence of steam pop in a VT ablation cohort using the STSF catheter, with 45 W. They reported a 1.2% incidence of audible steam pop. In the present study, we report a comparable safety profile, with a 0.02% incidence of steam pops among all ventricular applications. This suggests that QDOT ablation is associated with a favourable safety profile. It is important to note, however, that the settings in the aforementioned study were different. Prospective studies are needed in the future to assess the comparative incidence of steam pops.

### Standardized applications

In order to minimize the risk of steam pops, we aimed to reduce ablation duration to <60 s. The approach was not associated with a significant reduction in efficacy. As is the case for atrial ablation, where specific AI and CF values are targeted, we aimed to define standardized lesion parameters for ventricular ablation.^17^ It is our hypothesis that in most situations, 40 s of ablation with 50 W and a CF between 10 and 40 g is sufficient, if an impedance drop of 10 Ω is achieved and the impedance plateau phase is reached for applications in thin areas. Further systematic evaluation of these lesions is necessary in the future. For specific substrates, particularly septal and intramural substrates, longer applications were used. Prolonging ablation duration or changing the irrigation fluid both is predicted to increase the steam pop risk.

### Ablation for ventricular arrhythmia

In the present study, temperature-controlled ablation with QMODE 50 W ablation was safe for both VT and PVC ablation, without a decrease in effectiveness. An additional potential advantage of QMODE ablation is the promotion of low-flow 4 mL/min, possibly resulting in less fluid overload in patients with heart failure. We did not however investigate the impact on fluid balance and heart failure risk in the present study.^12^

### Benefits of QDOT ablation for ventricular arrhythmias

In the present study, we report that QDOT ablation is associated with a significant reduction in RF time without compromising efficacy or increasing the risk of complications. Based on the observation of a trend towards reduced procedure time, the shorter ablation time could positively influence procedure times. Systematic studies are needed to further investigate the impact on procedure time. The benefits of QDOT ablation in the ventricle extend beyond potential safety benefits. Micro-electrodes (ME) embedded in the catheter tip allows for more comprehensive characterization of the arrhythmogenic substrate. We previously reported that ME enhance distinction between near- and far-field signals and reduce the risk of AV block during para-Hisian PVC ablation (*Figures 1* and *3*).^14^ Micro-electrodes have also been reported to improve near-field EGM sensitivity, local abnormal ventricular activity (LAVA) annotation, signal-to-noise ratio, local activation timing, and substrate definition.^18–20^

### Catheter entrapment resolved using vHPvSD under constant traction

In one patient, the QDOT catheter was entrapped in the mitral valve apparatus. Multiple manoeuvres, including twisting, advancing, or applying constant traction, failed to retract the catheter. Eventually, the catheter was freed by simultaneously applying constant traction and ablating at 90 W for 4 s with a flow of 30 mL/min. We hypothesized that sudden local heating/oedema would increase the chances of catheter retraction. Previously reported approaches to retract catheters without causing damage to the valvular apparatus include real-time imaging and adenosine-induced ventricular asystole.^21,22^

## Conclusion

QMODE 50 W ablation for ventricular arrhythmia has a favourable safety profile without compromising effectiveness in patients undergoing ablation of ventricular arrhythmia.

### Limitations

This study has the inherent limitations of a non-randomized, single-centre prospective registry. Comparison was retrospectively performed for both VT and PVC ablation separately. In the retrospective dataset, data on steam pop are missing. Analysis of steam pops was limited to audible pops. Ablation time data from the multicentric PVC registry were not collected and are therefore not available for comparison. Since our application duration was typically limited to 40 s and all steam pop occurred in <40 s, it is unclear if our strategy could have prevented steam pop from occurring after ablating for a longer duration. Our findings do not add info on prolonged applications safety for the specific ablation mode explored.

## Data Availability

Derived data supporting the findings of this study are available from the corresponding author (B.B.) on request.
